# Transient numerical simulation of hemodynamics in bioprosthetic heart valves: insights into valve sizing and thrombosis risk

**DOI:** 10.1007/s11548-026-03577-4

**Published:** 2026-01-30

**Authors:** M. Berger, J. Golks, L. Gleissner, T. Senfter, Ch. Mayerl, N. Bonaros, C. G. Tepeköylü, L. Stastny, M. Grimm, M. Pillei

**Affiliations:** 1 Department of Medical Technologies, MCI - The Entrepreneurial School, Innsbruck, Austria; 2 Department of Industrial Engineering & Management, MCI - The Entrepreneurial School, Innsbruck, Austria; 3 Department of Cardiac Surgery, Medical University of Innsbruck, Innsbruck, Austria

**Keywords:** Bioprosthetic heart valves, Hemodynamics, Prosthesis–patient mismatch (PPM), Aortic valve replacement, Computational fluid dynamics (CFD)

## Abstract

**Purpose:**

This study aims to evaluate the hemodynamic effect of tilting after implantation of a bioprosthetic heart valve in an enlarged aortic annulus using computational fluid dynamics (CFD). The objective is to define the cost of excessive enlargement in transprosthetical flow and identify the optimal prosthesis size and implantation angle to reduce thrombosis and early degeneration.

**Methods:**

Virtual implantation of a 23-mm bioprosthetic model was conducted in a prespecified virtual aortic annulus of 23 mm. CFD simulations were conducted to analyze the hemodynamic parameters, including wall shear stress and turbulent kinetic energy, for bioprosthetic heart valves of the 23 mm (perpendicular implantation), 25 mm, and 27 mm (tilted implantation at different angles) after virtual annular enlargement. The simulations utilized transient flow models and mesh convergence studies, to ensure numerical accuracy and clinical relevance.

**Results:**

The 23-mm valve implanted without annular enlargement and in an aligned fashion to the annulus exhibited higher wall shear stress (WSS) and shear stress (SS) values than the 25-mm valve implanted at 12° tilted position and better hemodynamics than the 27-mm valve at 25° tilted position. The 25-mm valve after annular enlargement and implantation at 12° tilted position achieved the best hemodynamic performance together with a 23% and 7% reduction of WSS and SS as compared to the 23-mm valve.

**Conclusions:**

Slight oversizing and tilting after annular enlargement yields the best performance of an aortic bioprosthetic valve with lowest WSS and balanced turbulence.

## Introduction

Valvular heart disease (VHD) impairs blood flow and circulation, affecting ~ 13% of Europeans over 75, with aortic stenosis (AS) most common (~ 3.4%) [1]. AS, typically from calcific degeneration, restricts leaflet mobility, raising left ventricular outflow resistance and causing hypertrophy or systolic dysfunction. Aortic insufficiency (AI), from incomplete closure, leads to regurgitation, ventricular dilation, and progressive dysfunction [2].

The size of the aortic valve prosthesis implanted depends on the anatomical characteristics of the aortic annulus. The latter appears to be too small particularly in overweight patients with chronic arterial hypertension, thus limiting the size of the prosthesis which can be implanted. Implantation of a smaller aortic prosthesis than the one needed for the given body surface area results in prosthesis–patient mismatch [3]. This phenomenon has severe consequences, as implantation of an undersized prosthesis relative to the patient’s body surface area is associated with elevated transvalvular gradients and impaired long-term outcomes [3]. To mitigate this risk, surgeons may perform annular enlargement (AE) [4], a technique that increases the aortic root dimension and enables implantation of a prosthetic valve with a larger nominal diameter [3, 5–7]. AE procedures are technically demanding and often result in altered root geometry or non-standard implantation angles. In such cases, the ability to accommodate variable valve angulation or positioning flexibility could be of particular relevance, as it may facilitate optimal prosthesis seating and alignment within the modified anatomy.

A major advancement in CFD heart valve research is the use of patient-specific simulations from medical imaging. High-resolution MRI and CT provide detailed anatomy, enabling 3D models via segmentation [8]. Physiological boundary conditions, such as pressure and flow rates, can be obtained from ultrasound or MRI. Combining anatomical and hemodynamic data allows for more accurate, physiologically relevant simulations, improving CFD predictive value [9].

Fluid–structure interaction (FSI) models dynamically couple blood flow with valve motion by solving fluid and structural domains simultaneously. This approach captures the interaction between hemodynamics and tissue deformation, enabling realistic simulation of valve opening, closure, pressure distribution, and leaflet kinematics under physiological conditions [10]. FSI is essential for assessing valve durability, mechanical stress, and performance, as well as analyzing valvular repairs and pathologies. It helps evaluate transvalvular pressure and flow in stenosis and aids in optimizing repair techniques like edge-to-edge repair in insufficiency.

CFD is a key tool for analyzing hemodynamics in artificial heart valves. Studies focusing on aortic geometry, turbulence modeling, and flow parameters. James et al. [11] examined flow in functional and dysfunctional bileaflet valves using a test system simulating the aorta with extended entrance and outlet regions. Ranjith and Muraleedharan [12] simulated a 25-mm tilting disk valve under physiological pulsatile flow, modeling leaflet motion dynamically and validating results against experimental data. Blood was treated as a Newtonian fluid with constant viscosity, and the aorta was simplified as a cylindrical pipe with circular sinuses.

Shear stress (*τ*) is a key indicator of thrombotic risk in cardiovascular CFD studies, especially for artificial heart valves. Elevated *τ* levels between 10 and 50 Pa [13] are linked to increased platelet aggregation and thrombus risk. Wall shear stress (*τ*_wall_) above 16 Pa [13], particularly near the leaflet pivot and adjacent walls, may trigger platelet activation. High-velocity gradients from recirculating and swirling flows can further amplify these effects [11].

Surgeons use aortic AE to avoid PPM as it allows implantation of a valve with a larger nominal diameter. On the other hand, AE may lead to valve tilting as only one sector of the annulus is amenable to enlargement. Valve tilting may impact flow turbulence, shear stress, leaflet movement and affect the hemodynamic performance of the valve [14].

The aim of the study was to evaluate the hemodynamic effect of tilting after implantation of a bioprosthetic heart valve in an enlarged aortic annulus and to define the cost of excessive enlargement on transprosthetical flow. With one-way, and ultimately, two-way FSI coupling, the CFD–FEM simulations will enable the most realistic representation of the in vivo dynamic behavior of the Medtronic Avalus valve. Achieving this level of physiological fidelity represents the final objective of our ongoing research project, in which the simulation complexity is systematically increased in successive steps. As an essential starting point, fixed geometries are analyzed first. Accordingly, we initiated the investigation with a stationary CFD simulation using the same geometry presented in this study [15, 16]. Within this study, the complexity is increased by transient boundary conditions [17]. FSI is planned as future work and not included within this study.

## Methods

In the proposed investigation, the Medtronic Avalus heart valve sizes 23 mm, 25 mm, and 27 mm were investigated with transient boundary conditions to evaluate shear stress and wall shear stress. Therefore, the fluid domain is geometrically modeled and a mesh convergence study is performed. Finally, problematic volumetric and surface regions are evaluated. To assess the impact of different opening configurations on flow dynamics, two valve leaflet positions were considered.

The aortic geometry, including inlet and outlet extensions, was designed to ensure proper flow development and to minimize the influence of boundary conditions in the simulations. The inlet and outlet lengths were set to 400 mm and 270 mm, respectively, based on the findings of James et al. [9].

### Geometric modeling

**Fig. 1:**
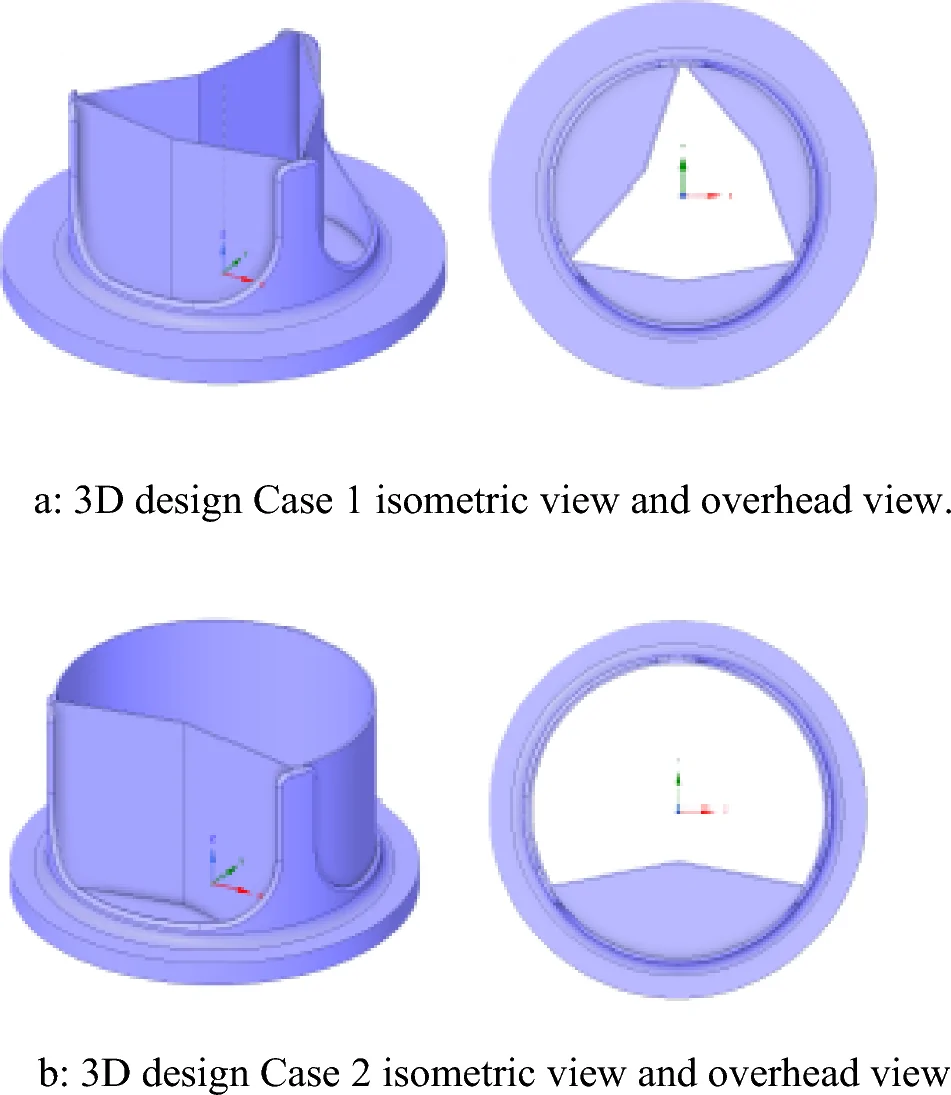
**a** 3D design Case 1 isometric view and overhead view. **b** 3D design Case 2 isometric view and overhead view

The geometry of the Medtronic Avalus bioprosthetic heart valve and the surrounding aortic root was modeled in Ansys Discovery. Three valve sizes (23 mm, 25 mm, and 27 mm) were evaluated, with the 23-mm valve serving as the baseline. The aortic root was modeled with an inlet length of 400 mm and an outlet length of 270 mm to allow for fully developed flow and minimize boundary condition effects. The Sinuses of Valsalva were included, with a diameter of 34 mm and a length of 24 mm, based on clinical recommendations. Two leaflet opening configurations were analyzed: Case 1, with moderate asymmetry (66%, 66%, and 33% opening), and Case 2, with one leaflet fully restricted (100%, 100%, and 33% opening). For the 25-mm and 27-mm valves, tilted implantation angles of 11.84° and 25.58°, respectively, were applied to fit the aortic geometry without altering the aortic wall (Fig. 1).

Case 1: Leaflets opened to 66%, 66%, and 33% of their full capacity.

Case 2: Leaflets opened to 100%, 100%, and 33% of their full capacity.

### CFD simulation

The computational domain was discretized using Ansys Meshing, with the physics preference set to CFD. Inflation layers were applied near the walls to resolve boundary layer effects, with five layers and a growth rate of 1.2. The element size was set to 3.9 mm, based on a mesh convergence study (see attachment).

Boundary conditions were defined to replicate physiological conditions:

*Inlet* A transient velocity profile [18] corresponding to a heart rate of 80 bpm was applied. The velocity data, generated using a Python script, were imported into Ansys Fluent as a time-dependent table.

*Outlet* A static pressure of 100 mmHg (13332.2 Pa) was applied, representing resting aortic conditions.

*Wall*: A no-slip boundary condition was imposed to simulate the interaction between blood and the vascular wall.

A glycerine-water mixture (51.37% glycerine by mass) was used as a blood analog, with a dynamic viscosity of *η* = 0.0035 Pa⋅s and a density of *ρ* = 1120.6 kg/m^3^. The Reynolds number, calculated as Re = 2209.18, indicated a transitional flow regime. The k-ω SST turbulence model was selected for its ability to capture near-wall effects and flow separation, critical for evaluating wall shear stress (WSS) and shear stress.

The simulations were performed in Ansys Fluent 2024R2 using the transient solver. The second-order upwind scheme was applied for spatial discretization of momentum, turbulent kinetic energy (TKE), and specific dissipation rate. Convergence criteria for residuals, including continuity and velocity components, were set to 1e−6. For the transient simulation, a time step size of 0.0025 s was used, with a total of 750 time steps per cardiac cycle.

Key hemodynamic parameters, including WSS and shear stress, were evaluated to identify regions of mechanical stress associated with thrombosis risk. WSS was directly extracted as a built-in output variable in Ansys Fluent, while shear stress was calculated using a custom field function (CFF) defined within the software. Isosurface visualizations were generated to highlight regions exceeding critical stress thresholds.

## Results

### Wall shear stress

**Fig. 2:**
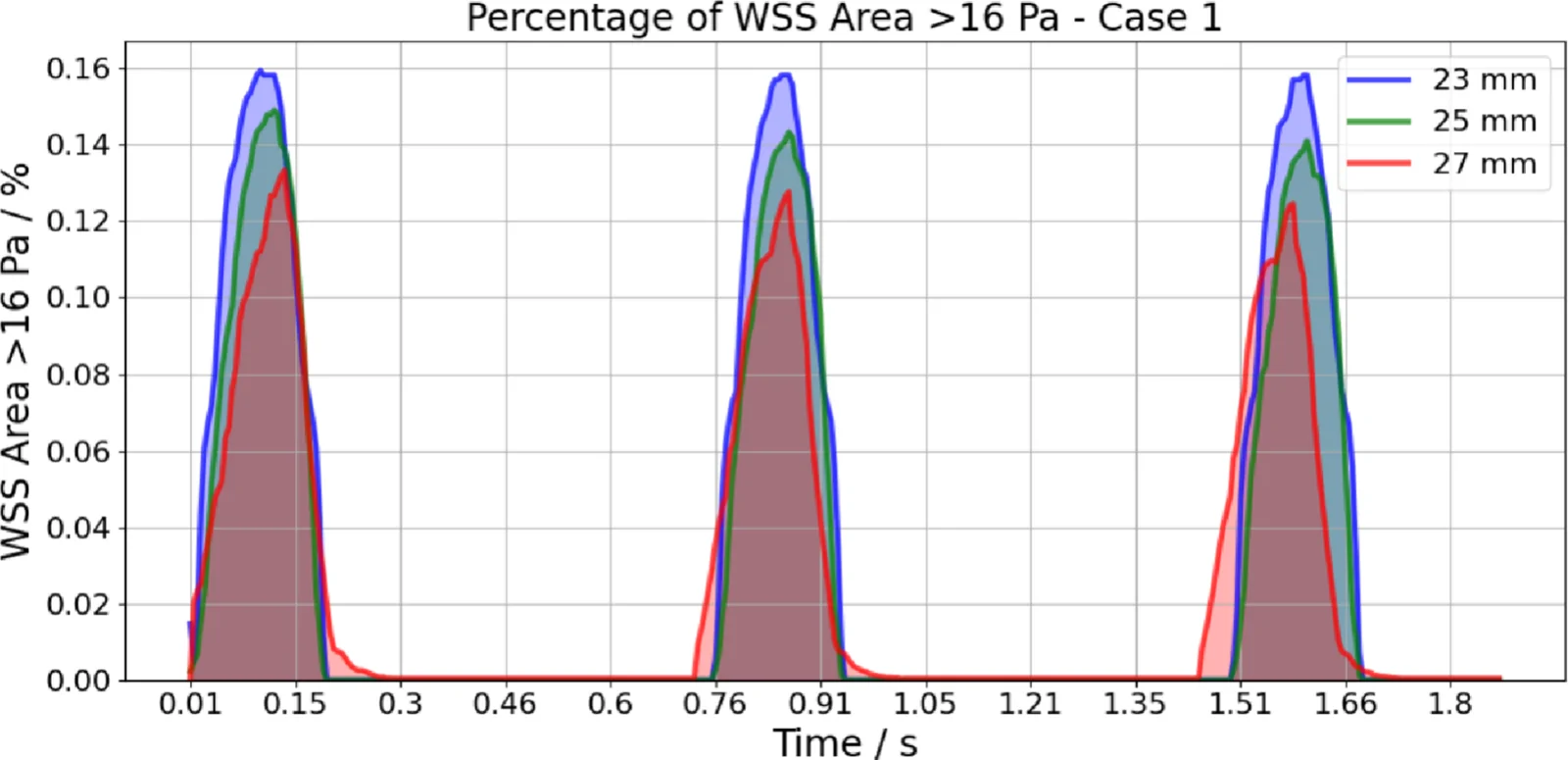
Percentage of WSS area bigger than 16 Pa for Case 1with respect to time, taken from [16]

**Fig. 3:**
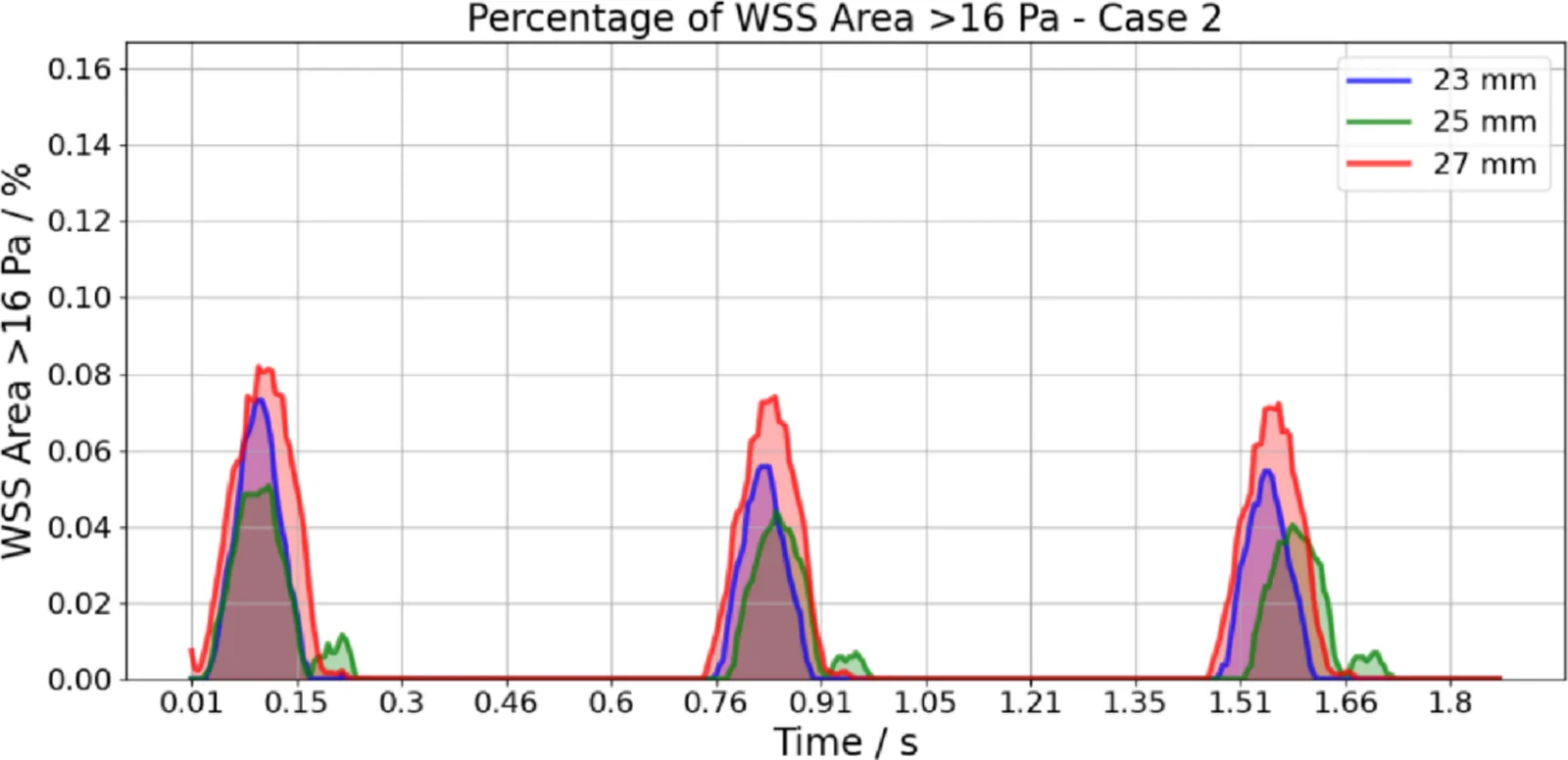
Percentage of WSS area bigger than 16 Pa for Case 2 with respect to time, taken from [16]

In Case 1, the highest wall shear stress (WSS) values are observed with the 23-mm valve, showing a peak of 0.1595% and an area under the curve (AUC) of 0.0592%·s. In contrast, the lowest values are found with the 27-mm valve, with a maximum of 0.1334% and an AUC of 0.0455%·s. A detailed comparison of peak and integrated WSS values across all valve sizes in both cases is provided in Table 1. The corresponding time-resolved curves are presented in Figs. 2 and 3.

**Table 1:** Comparison of AUC and maximum WSS > 16 Pa for different valve sizes in Case 1 and Case 2, taken from [17]

Valve size	AUC/%s		Max/%	
	Case 1	Case 2	Case 1	Case 2
23 mm	0.0592	0.0137	0.1595	0.731
25 mm	0.0498	0.0124	0.1491	0.0507
27 mm	0.0455	0.0229	0.1334	0.0818

Overall, Case 1 exhibits significantly higher wall shear stress (WSS) values compared to Case 2, with most measurements nearly doubled. In Case 2, the lowest WSS values are observed in the 25-mm valve, which additionally displays a secondary peak following the primary systolic pulse. In contrast, Case 1 shows the lowest WSS in the 27-mm valve, although the differences between valve sizes within this case are relatively small.

### Shear stress

**Fig. 4:**
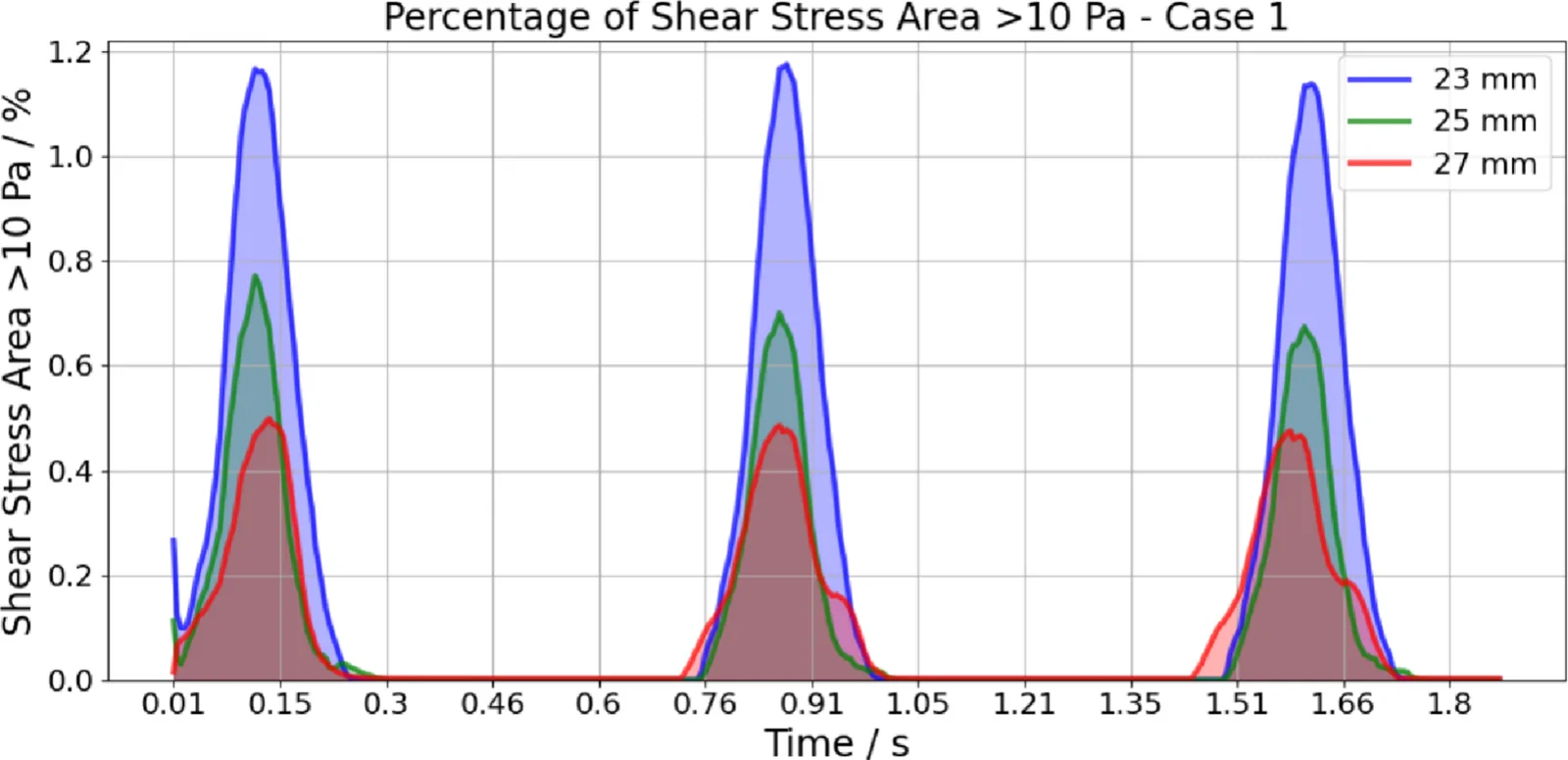
Percentage of shear stress bigger than 10 Pa for Case 1 with respect to time, taken from [17]

**Fig. 5:**
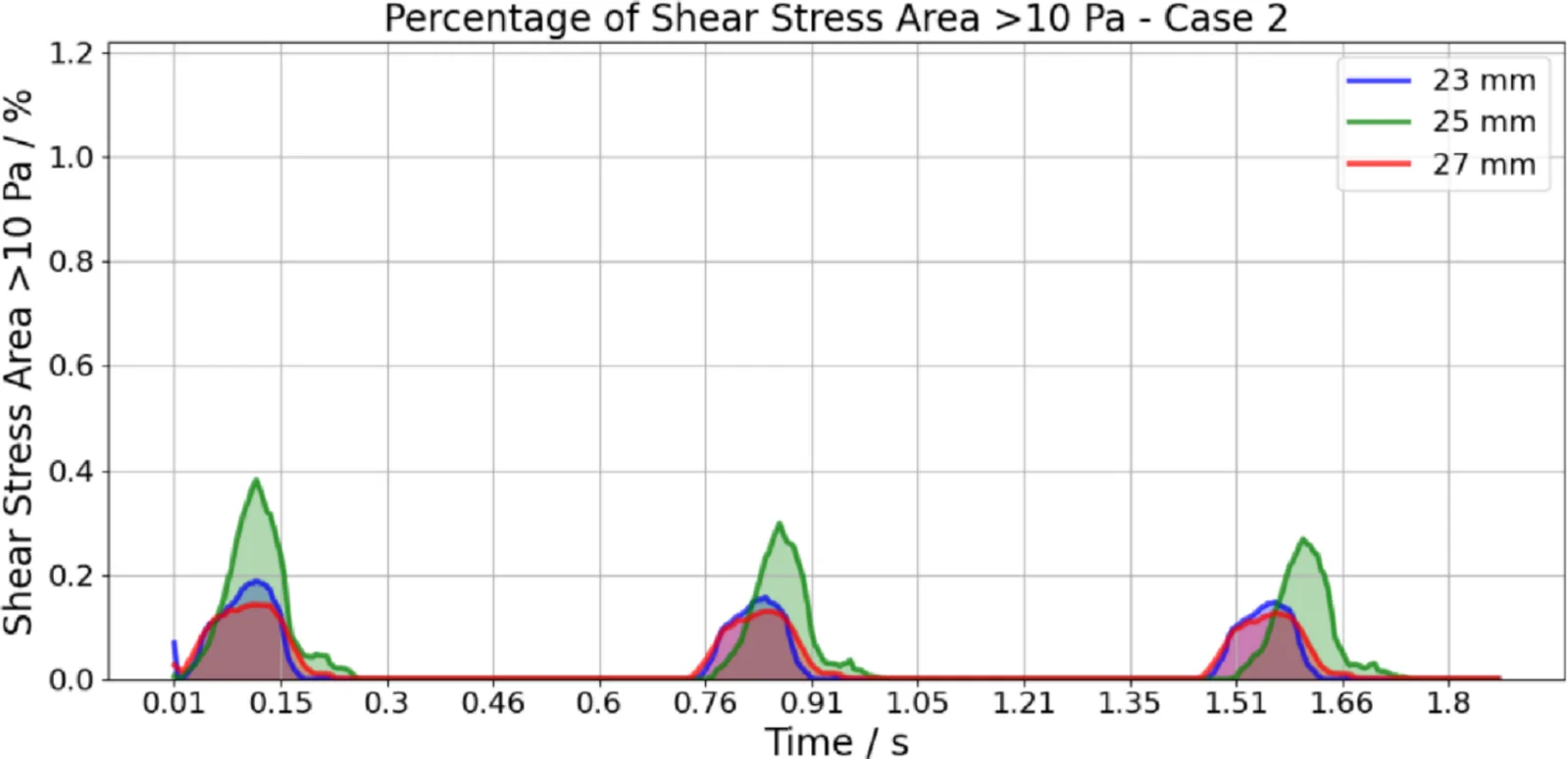
Percentage of shear stress bigger than 10 Pa for Case 2 with respect to time, taken from [17]

In Case 1, the highest shear stress values are observed in the 23-mm valve, with an area under the curve (AUC) of 0.3853%·s and a peak value of 1.1276%, which is more than twice the maximum recorded for the 27-mm valve (0.4995%). In Case 2, the highest shear stress is found in the 25-mm valve, while the 23-mm and 27-mm valves exhibit similarly low values. A detailed comparison of maximum and integrated WSS values for all valve sizes in both cases is provided in Table 2. The corresponding time-resolved curves are presented in Figs. 4 and 5.

**Table 2:** Comparison of the area under the curve (AUC) and maximum surface area exposed to shear stress > 10 Pa for different valve sizes in Case 1 and Case 2

Valve size	AUC / %s		Max / %	
	Case 1	Case 2	Case 1	Case 2
23 mm	0.3853	0.0477	1.1762	0.1883
25 mm	0.2063	0.0812	0.7723	0.3821
27 mm	0.1847	0.0511	0.4995	0.1430

In both cases, the lowest shear stress values are consistently observed in the 27-mm valve, with peak values of 0.4995% in Case 1 and 0.1430% in Case 2. In Case 2, the shear stress peak occurs later in the 25-mm valve compared to the 23-mm and 27-mm valves. In Case 1, the 27-mm valve exhibits a broader shear stress distribution at the beginning and end of the pulse, relative to the other valve sizes. Nevertheless, its overall AUC remains lower than that of the 23-mm and 25-mm valves. Overall, shear stress levels in Case 2 are significantly lower than those in Case 1.

### Wall shear stress visualization

**Fig. 6:**
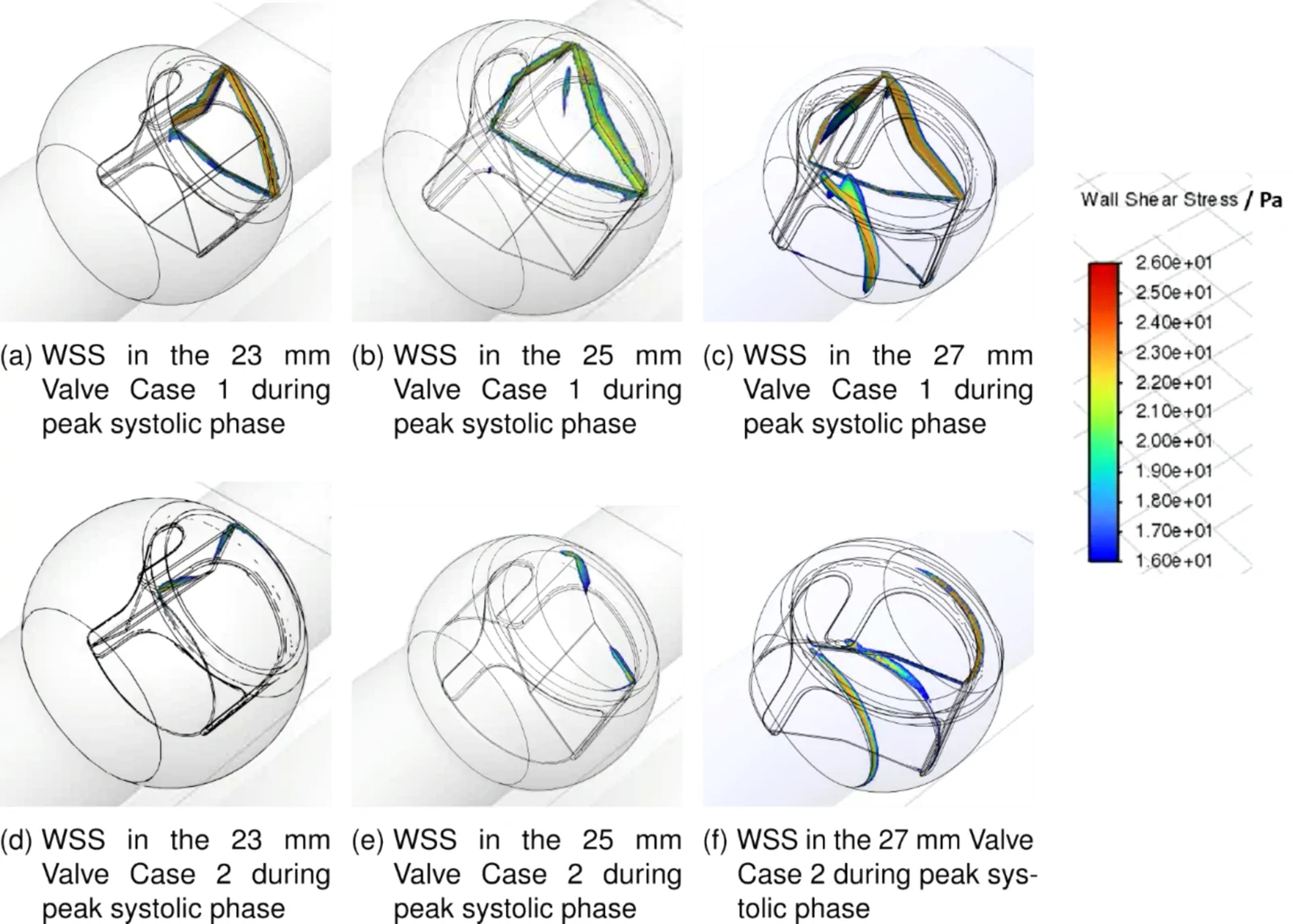
Wall shear stress (WSS) distributions for Case 1 (top row) and Case 2 (bottom row) are presented for three valve sizes (23 mm, 25 mm, and 27 mm). The WSS contours depict isosurfaces ranging from 16 to 26 Pa, highlighting regions of elevated shear stress. The corresponding color scale, in pascals, is shown on the right. Flow direction in all visualizations is from the backward right to the forward front. Taken from [17]

In the 23-mm valve (Case 1), medium to high wall shear stress (WSS) occurs along the valve’s first curvature (Fig. 6a), while Case 2 shows WSS mainly on the restricted side with minimal levels elsewhere (Fig. 6d). For the 25-mm valve, Case 1 displays moderate WSS across the entire valve curvature (Fig. 6b), whereas Case 2 confines WSS to the restricted side with lower values near the leaflet peak (Fig. 6e). In the 27-mm valve (Case 1), WSS is less intense but present along the valve’s initial and final curvatures and adjacent walls during systole (Fig. 6c). Case 2 shows WSS concentrated on one side but spread over more curvatures with overall lower intensity (Fig. 6f).

In the 25-mm valve (Case 2), an area at the junction of the two fully opened leaflets near the valve outlet is subjected to WSS shortly after the peak of the systolic phase. In the 27-mm valve (Case 1), localized regions of WSS persist throughout the entire diastolic phase.

### Shear stress visualization

**Fig. 7:**
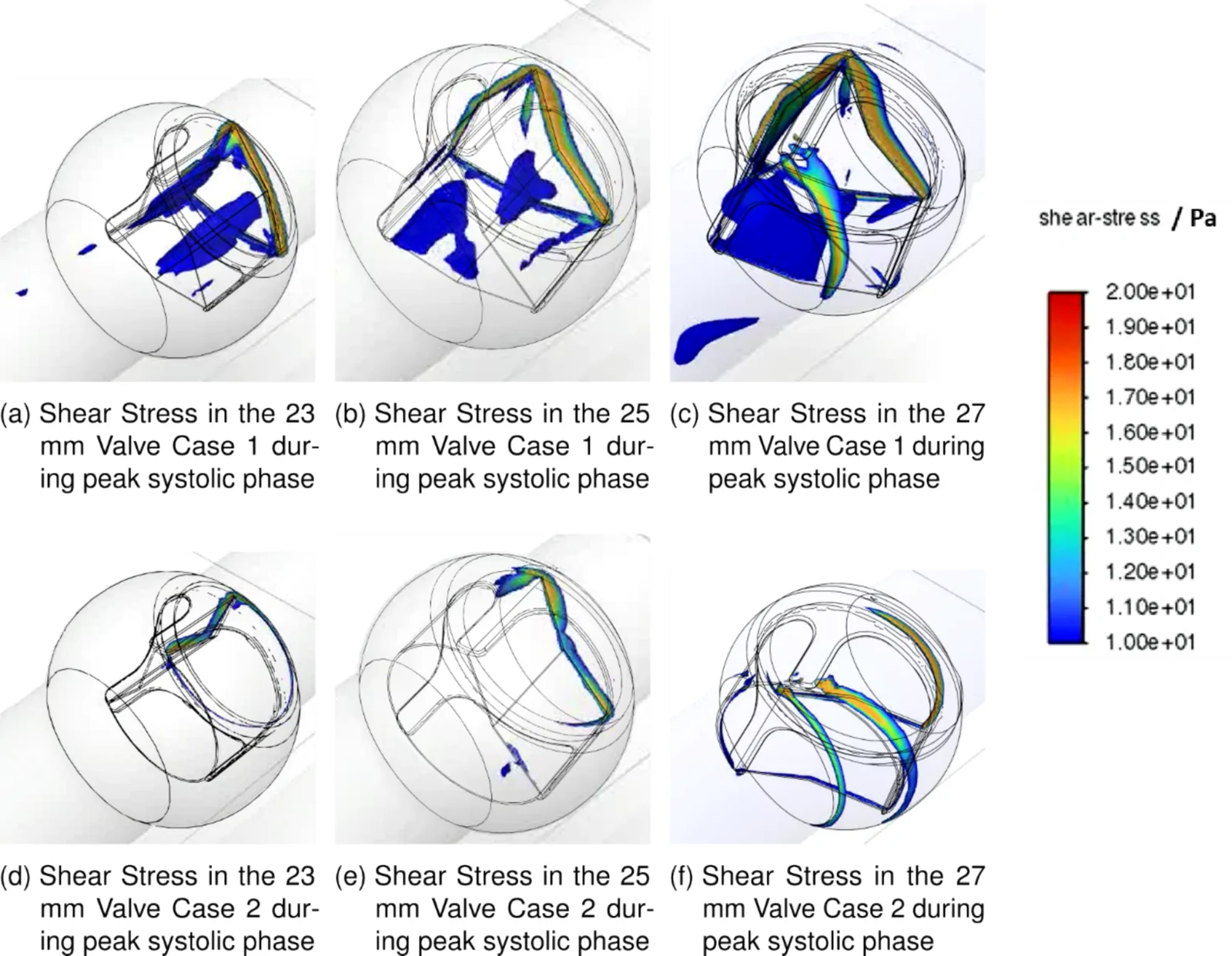
Shear stress distributions for Case 1 (top row) and Case 2 (bottom row) are presented for three valve sizes (23 mm, 25 mm, and 27 mm). The shear stress contours depict isosurfaces in the range of 10 Pa to 20 Pa, highlighting regions of elevated shear stress. The corresponding color scale, in pascals, is displayed to the right. Flow direction in all visualizations is from the backward right to the forward front. Taken from [17]

In Case 1, the 23-mm valve shows shear stress along the outer curvature in the first frame of the cardiac cycle, as illustrated in Fig. 7a. In contrast, Case 2 exhibits a markedly smaller region of shear stress, limited to the initial contact area where the flow meets the curvature (Fig. 7d). Furthermore, downstream of the curvature, shear stress levels are noticeably lower in Case 2.

For the 25-mm valve in Case 1, the shear stress distribution along the first curvature resembles that of the 23-mm valve in the same case. However, a more extensive region of lower shear stress is observed immediately downstream of the curvature, along the valve walls, as shown in Fig. 7b. In Case 2, shear stress increases distinctly along one side of the entire bend as the flow progresses through the valve. Additionally, regions of shear stress are visible on the valve walls shortly after flow entry, as illustrated in Fig. 7e.

In the 27-mm valve (Case 1), shear stress is observed at both the initial and final curvatures of the valve. In these regions, higher shear stress occurs along the upper sections of the curvature, while lower shear stress is distributed along the valve walls, as shown in Fig. 7c. In Case 2, the overall shear stress distribution is similar, but the affected areas are smaller. Shear stress remains present at both the inlet and outlet regions of the curvature, with more pronounced values along the upper portions of the bend. Minimal shear stress is observed along the valve walls, as illustrated in Fig. 7f.

**Fig. 8:**
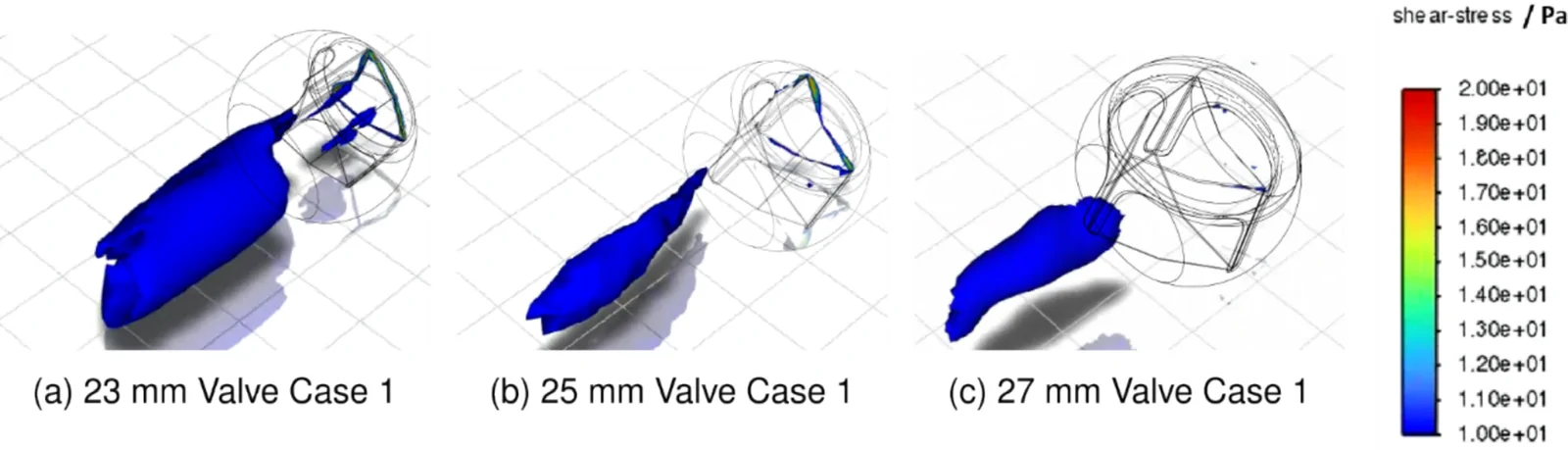
Shear stress distribution after the peak systolic phase for different valve sizes in Case 1. The corresponding color scale, in pascals, is shown on the right. Flow direction is from the backward right to the forward front

In Case 1 of the 23-mm valve, extensive regions of shear stress are observed in the aorta downstream of the valve shortly after the systolic peak. A similar pattern is seen in the 25-mm valve (Case 1), although the affected area is smaller and exhibits a more asymmetrical distribution, concentrated on one side. In the 27-mm valve (Case 1), the extent of shear stress is further reduced, with only small, localized regions of shear stress visible along the valve edges throughout the diastolic phase. This localized diastolic shear stress is also present in Case 2 of the 27-mm valve. Figure 8 illustrates the shear stress distributions for all Case 1 scenarios shortly after the peak of the systolic phase.

### Turbulent kinetic energy

**Fig. 9:**
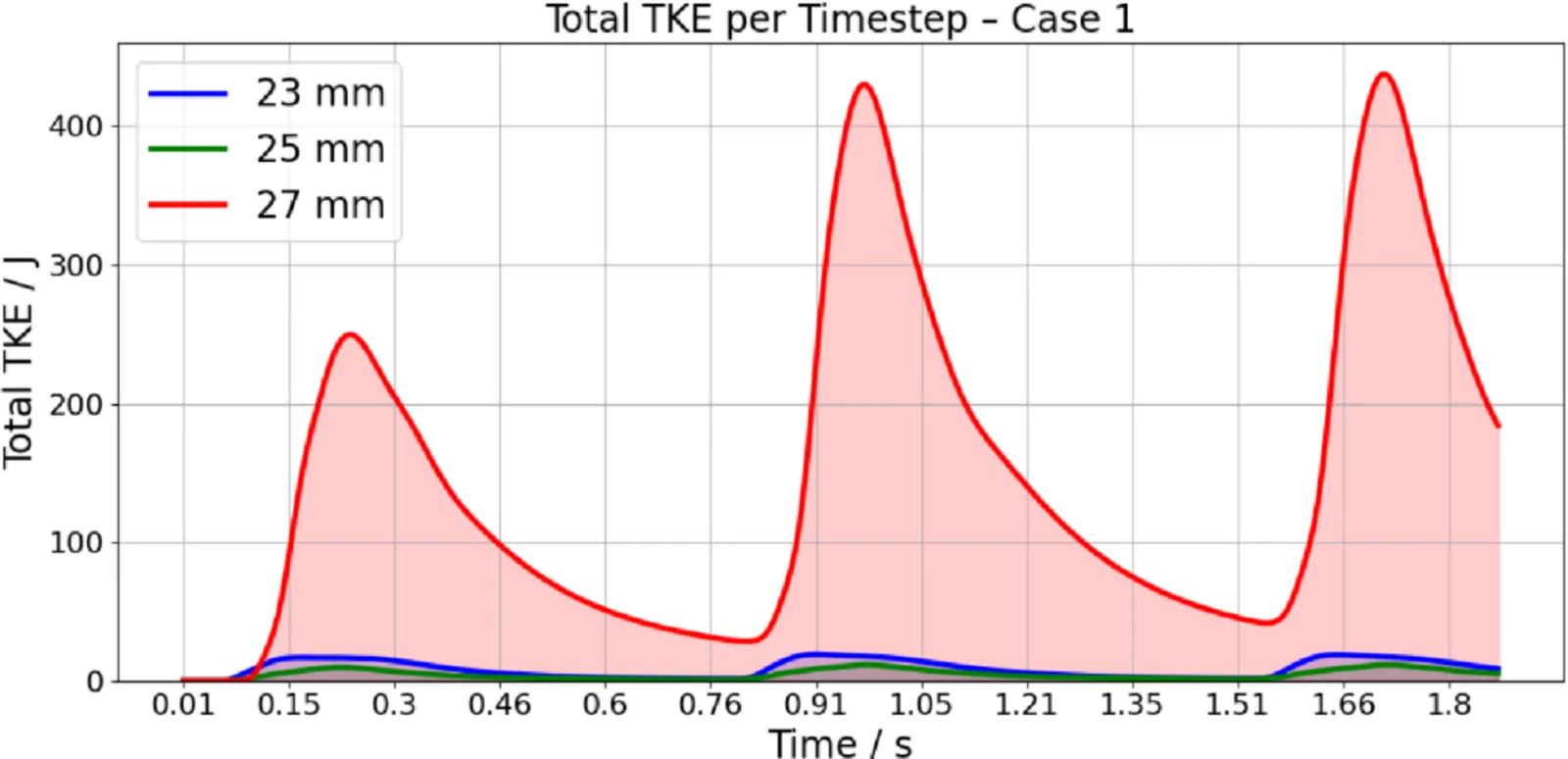
Total volume integrated TKE with respect to time for Case 1. Taken from [17]

**Fig. 10:**
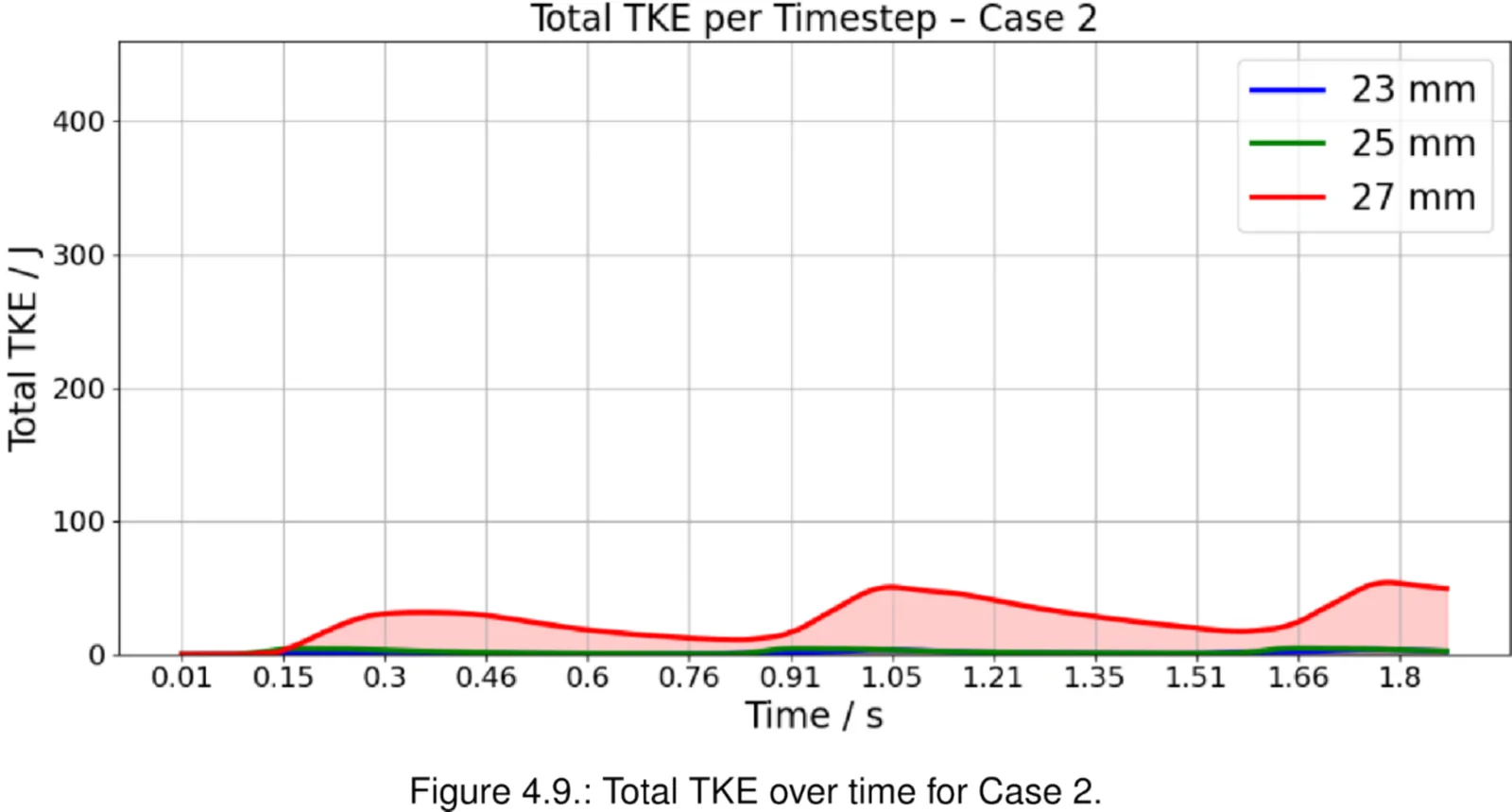
Total volume integrated TKE with respect to time for Case 2. Taken from [17]

In terms of total turbulent kinetic energy (TKE) integrated with respect to the volume, the 27-mm valve exhibits the highest values in both cases, with an area under the curve (AUC) of 276.48 J·s in Case 1 and 49.11 J·s in Case 2. In Case 1, the 25-mm valve shows the lowest values in both AUC. In Case 2, the 27-mm valve yields the lowest values, although the difference compared to the 25-mm valve is minimal. A detailed comparison of maximum and AUC values of total TKE across all valve sizes and cases is provided in Table 3. The corresponding time-resolved curves are shown in Figs. 9 and 10.

**Table 3:** Comparison of area under the curve (AUC) and maximum total turbulent kinetic energy (TKE) values for different valve sizes in Case 1 and Case 2

Valve size	AUC/Js		Max/J		Max TKE per volume/(J/m^3^)	
	Case 1	Case 2	Case 1	Case 2	Case 1	Case 2
23 mm	16.41	2.79	19.15	3.76	78.25	23.38
25 mm	8.60	3.95	11.58	4.59	68.72	45.46
27 mm	276.48	49.11	437.91	54.22	58.92	19.95

AUC and MAX is based on volume integrated turbulent kinetic energy. Similar results of max per volume in J/m^3^ could be found in [19–21]

In Case 2, the values are notably lower than in Case 1, and for the 27-mm valve, the TKE never drops to zero throughout the measurement period.

**Fig. 11:**
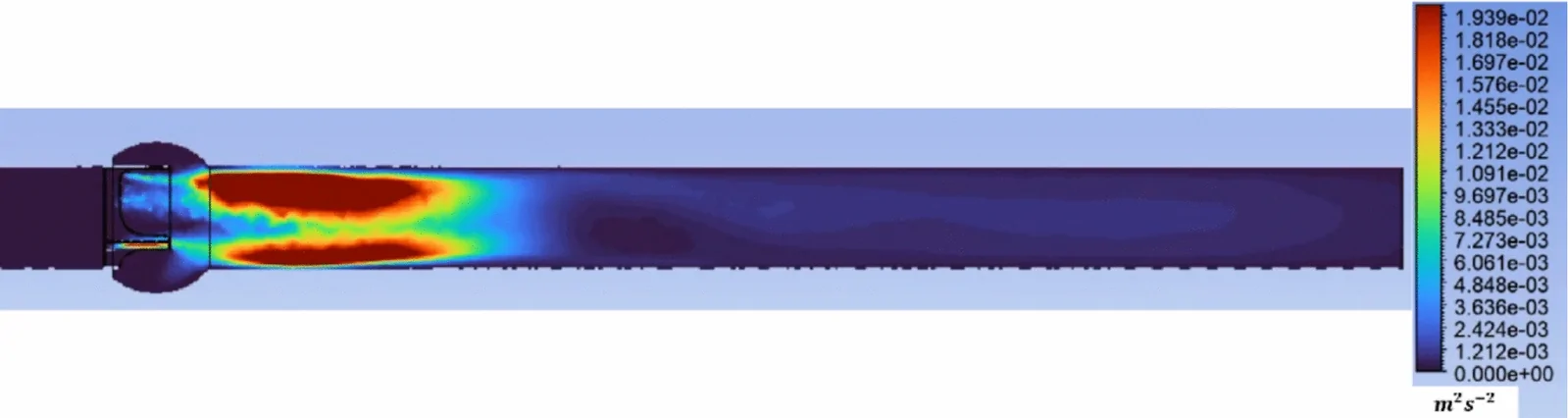
Visualization of turbulent kinetic energy (TKE) distribution in m^2^/s^2^. Flow direction is from left to right. Taken from [17]

To better understand the regions of elevated turbulent kinetic energy (TKE), a visualization using the 23-mm valve is presented in Fig. 11. The figure illustrates that higher TKE values are primarily concentrated near the wall regions and on a small area of the valve surface. No TKE is detected upstream of the valve, whereas increased TKE persists downstream, well beyond the valve.

### Velocity vector field

**Fig. 12:**
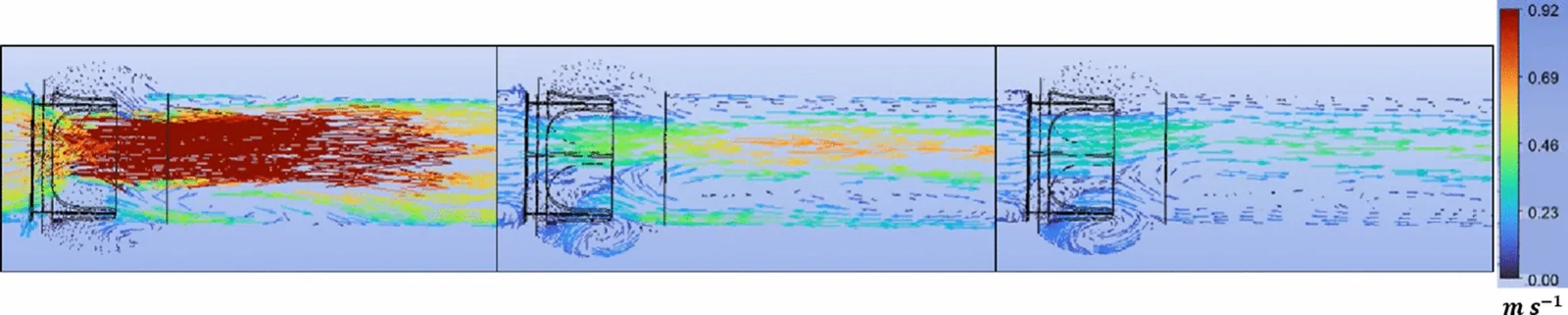
Velocity profile of the 23-mm valve in Case 1. Flow direction is from left to right. Taken from [17]

For the 23-mm valve, the highest velocities are consistently concentrated in the central region of the aorta across all time steps. As illustrated in Fig. 12, a small vortex forms immediately downstream of the valve in the lower region, as well as within the lower section of the sinuses of Valsalva.

**Fig. 13:**
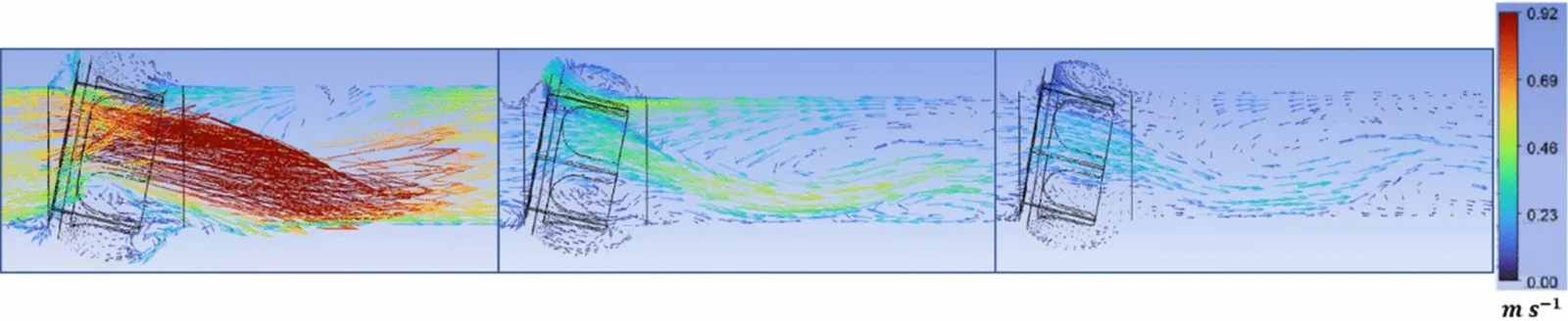
Velocity profile of the 25-mm valve in Case 1. The flow direction is from the left to the right. Taken from [17]

In the case of the 25-mm valve, the fluid is initially deflected toward the lower region of the aorta downstream of the valve before redirecting back toward the center. Simultaneously, a large vortex develops in the upper region of the aorta, directly above the main flow stream, extending up to the sinuses of Valsalva. As the flow returns toward the centerline, a smaller vortex forms in the lower region of the aorta. Conversely, reduced fluid flow is observed in the lower part of the sinuses compared to the upper part (Fig. 13).

**Fig. 14:**
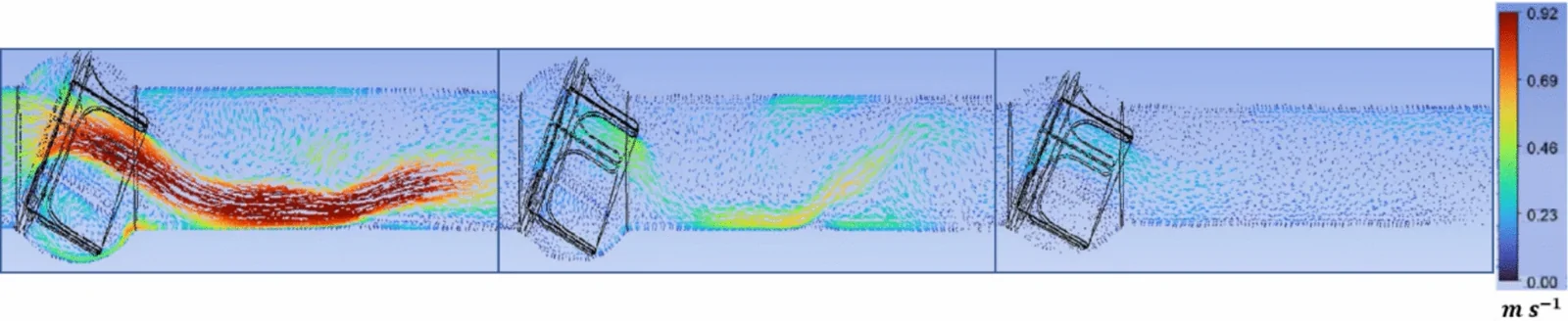
Velocity profile of the 27-mm valve in Case 1. The flow direction is from the left to the right. Taken from [17]

For the 27-mm valve, the highest velocities are located in the upper-central region and are immediately deflected toward the lower part of the aorta. A vortex forms along the path of this deflected jet. As the flow progresses, the high-velocity stream shifts upward again from the lower region toward the upper part of the aorta. This is illustrated in Fig. 14.

**Fig. 15:**
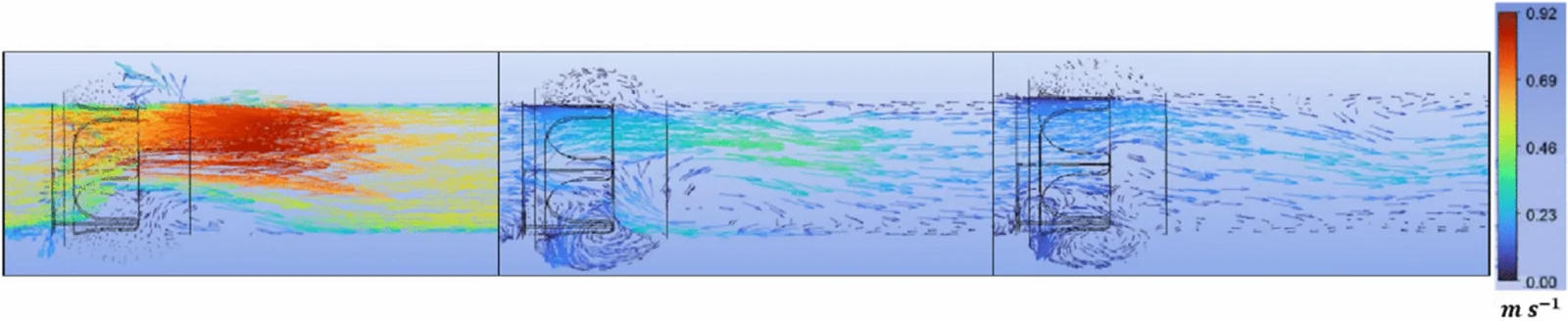
Velocity profile of the 22 mm valve in Case 2. The flow direction is from the left to the right. Taken from [17]

In the 23-mm valve model, higher flow velocities are observed in the upper region of the aorta during the peak systolic phase. As the flow progresses, the main jet of elevated velocity shifts back toward the central axis of the aorta. This movement is accompanied by the formation of vortices both in the upper region of the aorta and immediately downstream of the valve in the lower region. Minimal flow activity is detected within the lower part of the sinuses of Valsalva. These flow characteristics are illustrated in Fig. 15.

**Fig. 16:**
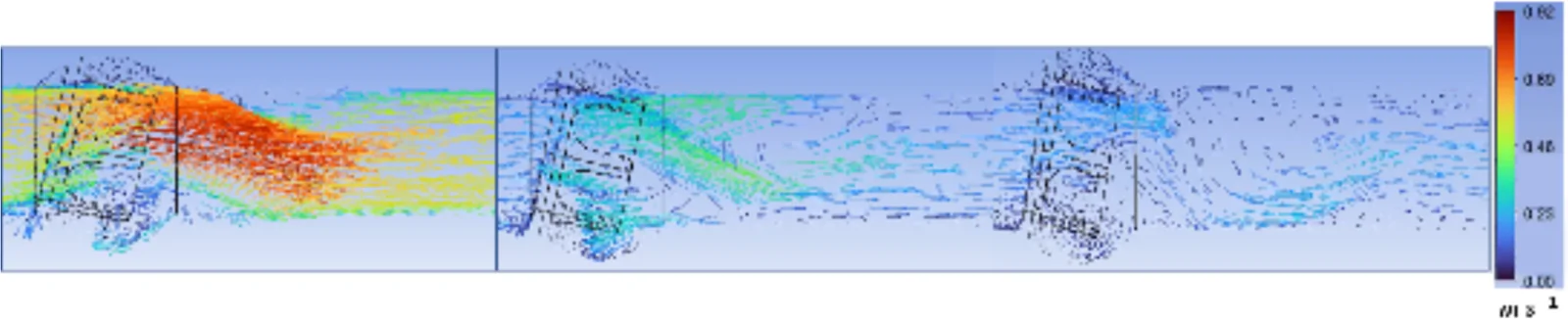
Velocity profile of the 25-mm valve in Case 2. The flow direction is from the left to the right. Taken from [17]

For the 25-mm valve, regions of high velocity are initially concentrated in the upper area near the valve but are rapidly redirected toward the central and lower regions of the aorta. As the flow develops, vortices form in the midsection of the aorta and immediately downstream of the valve in the lower region, extending into the sinuses of Valsalva. Over time, the main flow shifts toward the lower part of the vessel before returning to a more central position. This is illustrated in Fig. 16.

**Fig. 17:**
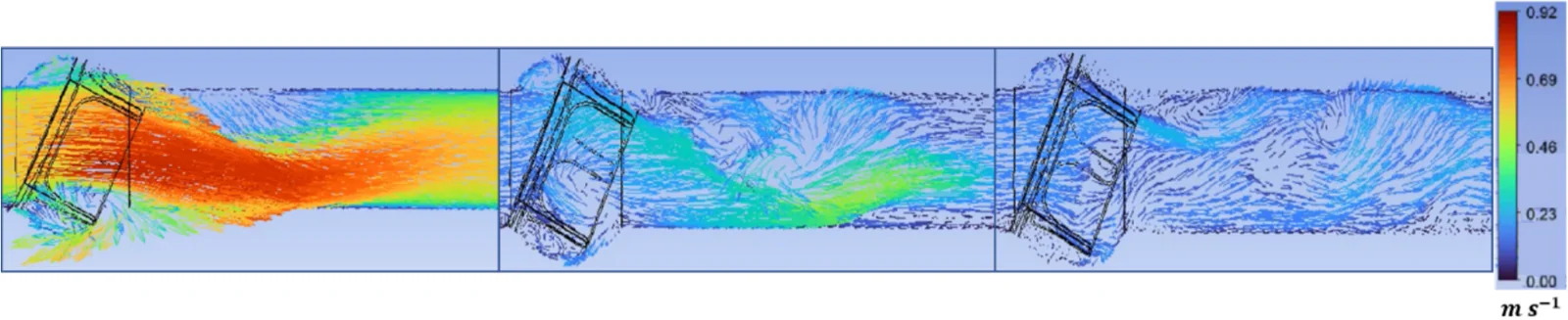
Velocity profile of the 27-mm valve in Case 2. The flow direction is from the left to the right. Taken from [17]

For the 27-mm valve, the highest velocities initially concentrate in the central region of the flow. They are subsequently deflected toward the lower part of the aorta before returning to a more central position. As the flow evolves, vortices begin to form in the upper region of the aorta. Over time, additional vortex structures develop throughout the entire aortic section, as illustrated in Fig. 17.

## Discussion

Elevated WSS in Case 1 for the 23-mm valve likely results from the flow jet directly hitting the aortic wall without deflection, with high jet velocity amplifying shear stress. A slight valve tilt could reduce WSS by redirecting flow. In Case 2, the tilted 27-mm valve directs the jet more strongly toward the wall, causing higher WSS. For the 25-mm valve (Case 2), a large vortex above the main jet, caused by tilt, redirects flow back toward the valve, creating a secondary WSS peak that may indicate a critical stress zone. Overall, the 27-mm valve shows the lowest total WSS and shear stress except in Case 2, where tilt and restricted opening increase flow deflection and WSS near the valve. The 25-mm valve in Case 2 shows the best WSS but highest peak shear stress, likely due to unfavorable tilt and partial opening. Elevated turbulent kinetic energy (TKE) contributes to increased shear and WSS, potentially damaging the aortic wall and blood components [22]. The 25-mm valve in Case 2 has the highest with shear stress, while the 23-mm valve consistently shows the highest TKE overall. Case 1 exhibits higher hemodynamic stress across all valve sizes, marking it as the more critical condition.

Visualizations reveal that the valve's initial section bears the highest mechanical load (WSS and shear stress), suggesting reinforcement there may be useful. Greater valve tilt and size spread shear stress more broadly downstream, though over a smaller surface area—indicating a trade-off between localized and distributed stress. All valves show reduced velocities in the upper sinuses of Valsalva, a potential site for thrombus formation. In tilted valves, especially the 25 mm (Case 2), slow flow in front and below the valve suggests stagnation risk. In contrast, the 27-mm valve with greater tilt shows higher velocities in these zones, raising the risk of blood damage. Case 1 of the 27-mm valve shows a focused, high-speed jet from the restricted tilt, impacting the wall and increasing stress during stronger pulses. Across all valves, Case 1 exhibits higher velocities due to narrower openings. Without moving leaflets, pressure differences were minimal, though increased tilt slightly raised downstream pressure.

Salman et al. observed similar velocity patterns, including a vortex in the sinuses of Valsalva and a central high-velocity region. They also noted an upstream vortex after the pulse phase. Their WSS analysis showed highest values at edges of valves with reduced opening, but tilted valve configurations were not examined [23].

James et al. studied malfunctioning bileaflet valves with one leaflet open and the other nearly closed. They observed similar flow deflection toward the aortic wall and increased velocities in the deflected stream, comparable to tilted valves in our study. Both showed intensified vortex formation near the wall and valve region, aligning with our results [11].

Literature reports peak turbulent kinetic energy (TKE) values between 0.9 and 4.8 mJ [24]. In this study, all Case 1 valves exceeded this range, likely due to tilted implantation and smaller effective orifices causing higher flow velocities and increased TKE. In Case 2, the 27-mm valve stayed within the reported range, while the 23-mm valve slightly exceeded it, probably due to its smaller size. Elevated TKE in the 25-mm valve of Case 2 may result from abnormal tilt.

Multiple studies have investigated pressure contours in patients with calcified and healthy valves, reporting elevated pressures downstream of the valve in calcified cases. Similarly, the present study found that smaller valve openings result in higher post-valve pressure [23, 25]. In this study, the valve tilt reduces the effective opening area available for pressure equalization, leading to increased pressure gradients in tilted valves compared to non-tilted configurations.

AE represents a well-recognized surgical strategy to prevent PPM by permitting implantation of a larger valve prosthesis [3, 5–7]. While AE can significantly improve postoperative hemodynamics, it frequently alters the aortic root geometry, resulting in asymmetric expansion or partial distortion of the annular plane. This is related to the fact that AE can only be performed at the non-coronary sinus, while the implantation site at the left and right coronary sinus is anatomically defined by the presence of the coronary ostia. One-sided enlargement provides the opportunity to implant an oversized valve by increasing the height of implantation at the non-coronary sinus, thus changing the perpendicularity of the valve to the ventricular outflow. Consequently, the prosthesis is often implanted at a non-orthogonal orientation relative to the left ventricular outflow tract. Under such conditions, valve tilt or angulation becomes clinically relevant, as it may influence local flow acceleration, shear stress distribution, and turbulence. This study provides a computational assessment of these effects, demonstrating that moderate tilt following AE can improve hemodynamic performance by reducing wall shear stress and enhancing flow uniformity, whereas excessive tilt may induce flow asymmetry and elevated shear stress zones. These findings underline that the ability to control valve positioning in enlarged annuli may be of genuine surgical relevance—particularly in AE procedures, where geometric distortion cannot always be avoided. Thus, the proposed simulation framework contributes to a better mechanistic understanding of flow alterations in anatomically modified annuli and supports optimization of prosthesis implantation strategies in AE contexts.

With our approach, we are able to assess the limitations of the simplified study and to what extent more sophisticated FSI models (future study), with their considerable computational demands. The presented study therefore represents an important intermediate step, allowing us to determine whether CFD simulations with fixed geometries, transient boundary conditions, and transient simulation can already provide valuable insights. Ultimately, this comparison will help to define the balance between model simplicity and physiological accuracy, indicating how even less computationally demanding simulations can contribute to improved understanding and surgical optimization of blood flow within the aortic valve. It should be noted that the WSS and shear stress values presented in this study were computed directly from the Reynolds-averaged flow field. Consequently, the influence of turbulent fluctuations on the local shear stresses was not captured. These fluctuations can contribute to the total shear stress, potentially exceeding the magnitude of the Reynolds-averaged component. Therefore, the reported WSS values should be interpreted with this limitation in mind. Simulations based on LES and DNS could give further insights with much higher computational effort. We used a water–glycerine mixture to facilitate experimental flow analysis while avoiding the use of blood. This approach allows for the implementation of advanced measurement techniques such as particle image velocimetry (PIV), laser doppler anemometry (LDA), or high-speed imaging in future studies. By employing this method, we can directly compare the velocity vector fields obtained from experiments with those predicted by numerical simulations, enabling a robust validation of our computational models.

## Conclusion

The results suggest that selecting an intermediate or larger valve size, such as the 25-mm or 27-mm configuration with a tilt, may be preferable for improving flow conditions, reducing damaging shear stress, and minimizing the risk of thrombosis. However, increased tilt angles lead to a broader distribution of shear stress and WSS within the valve, as well as elevated TKE values, which could contribute to energy loss and potential damage to the aortic wall.

In Case 1, representing the more critical scenario with generally higher stress levels, the 27-mm valve demonstrates the most favorable hemodynamic performance. This indicates that combining a larger valve size with tilting may synergistically enhance flow characteristics. Further investigation is warranted to explore these interactions more comprehensively.

## Figures and Tables

**Fig. 18:**
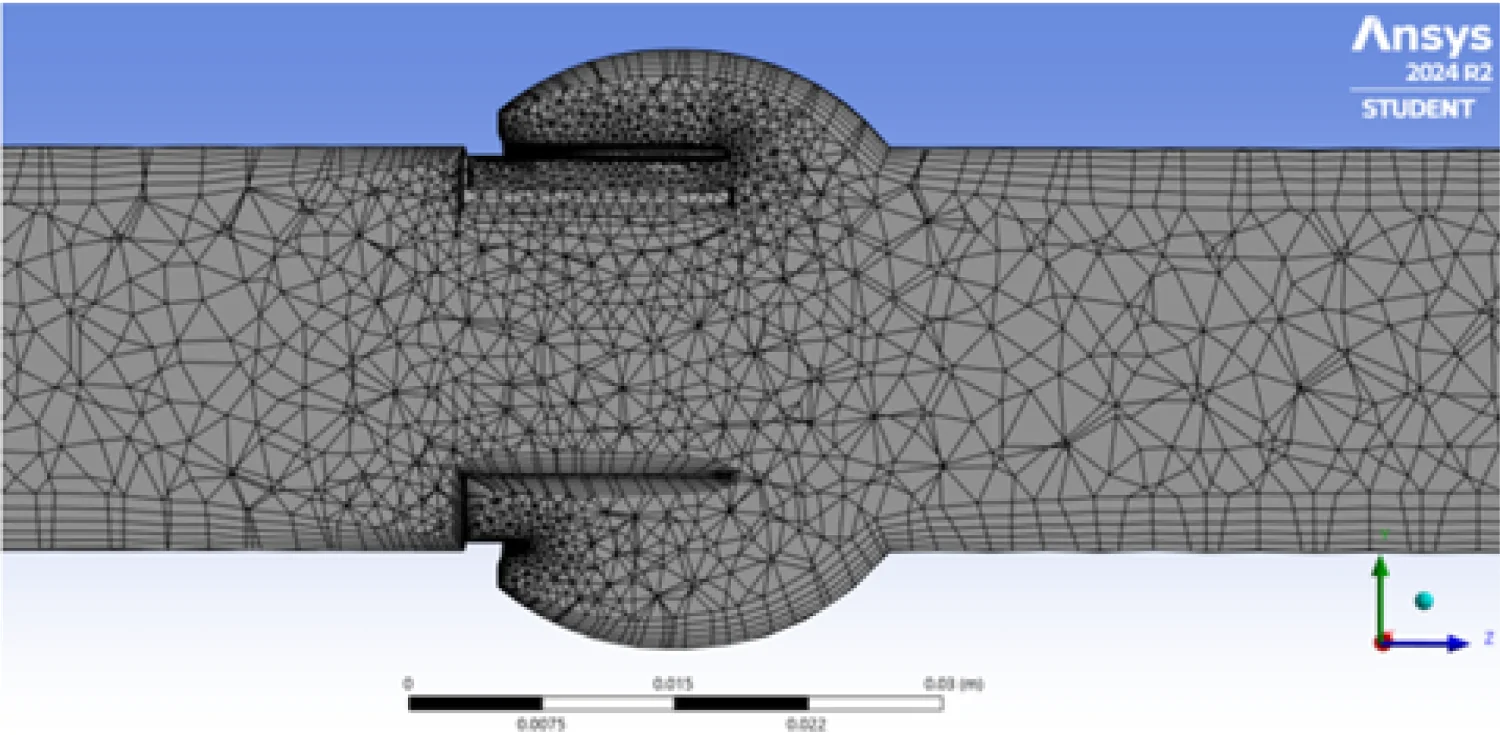
Generated mesh for the valve and aortic geometry. Visible inflation layers improve near-wall accuracy, while refined edge elements capture geometric details

## Appendix

The grid convergence index (GCI) was calculated to assess numerical error and verify mesh convergence. A safety factor of Fs = 1.25 was applied, and the relative error e between two mesh resolutions was used to compute the GCI. The calculated AR values are 1.00210 for Vmax and 1.01410 for TKEmax. Accordingly, a mesh size of 3.9 mm was chosen for all simulations (see Fig. 18 and Table 4).

**Table 4:** Results of mesh convergence study based on [26]

Mesh size	Vmax in m/s	TKEmax in m^2^/s^2^
1.95	0.39948	0.00533653
3.9	0.39864	0.00526232
7.8	0.39395	0.00472059
